# The Interplay Between Cellular Senescence and Lipid Metabolism in the Progression of Metabolic Dysfunction-Associated Steatotic Liver Disease (MASLD)

**DOI:** 10.3390/ijms27021066

**Published:** 2026-01-21

**Authors:** Eleftheria M. Mastoridou, Anna C. Goussia, Agapi Kataki, Efthymios Koniaris, Georgios K. Glantzounis, Alexandra Papoudou-Bai, Panagiotis Kanavaros, Antonia V. Charchanti

**Affiliations:** 1 Department of Anatomy-Histology-Embryology, Faculty of Medicine, School of Health Sciences, University of Ioannina, 45110 Ioannina, Greece; e.mastoridou@uoi.gr (E.M.M.); pkanavar@uoi.gr (P.K.); 2 Department of Pathology, Faculty of Medicine, School of Health Sciences, University of Ioannina, 45110 Ioannina, Greece; agoussia@uoi.gr; 3 Laboratory of Surgical Research, First Department of Propaedeutic Surgery, Hippocration Hospital of Athens, Athens Medical School, National and Kapodistrian University of Athens, 11528 Athens, Greece; akataki@med.uoa.gr; 4 Department of Pathology-Anatomy, Hippocration Hospital of Athens, Athens Medical School, National and Kapodistrian University of Athens, 11528 Athens, Greece; e.koniaris@hippocratio.gr; 5 HPB Unit, Department of Surgery, University General Hospital of Ioannina, School of Medicine, University of Ioannina, 45110 Ioannina, Greece; gglantzounis@uoi.gr; 6 Pathology Department, German Medical Institute (GMI), Limassol 4040, Cyprus; alexandra.papoudou@goc.com.cy

**Keywords:** cellular senescence, lipid droplets, metabolic dysfunction-associated steatotic liver disease, senescence-associated secretory phenotype, mitochondrial dysfunction, fatty acid oxidation

## Abstract

Metabolic dysfunction-associated steatotic liver disease (MASLD), previously known as non-alcoholic fatty liver disease (NAFLD), is now recognized as the leading cause of chronic liver disease worldwide. MASLD spans a spectrum ranging from simple steatosis to metabolic dysfunction-associated steatohepatitis (MASH) and is linked to progressive fibrosis and ultimately hepatocellular carcinoma (HCC). Growing evidence implicates cellular senescence (CS) and lipid droplets (LDs) as key drivers of disease progression, although their interaction remains poorly characterized. This review provides an integrative and stage-dependent synthesis of current mechanistic insights into how bidirectional crosstalk between CS and LD regulation shapes the transition from steatosis to MASH. Senescent hepatocytes display altered lipid metabolism, including upregulation of receptors such as cluster of differentiation (CD) 36, enhancing lipid uptake to meet increased energy demands. Initially, elevated free fatty acid influx can activate peroxisome-proliferator-activated receptor alpha (PPARα), promoting fatty acid oxidation (FAO) as a compensatory response. Over time, persistent CS under steatotic conditions leads to mitochondrial dysfunction and suppression of fatty acid oxidation (FAO), while the senescence-associated secretory phenotype (SASP), largely driven by nuclear factor—kappa B (NF-κB) signaling, promotes chronic hepatic inflammation. By framing LDs as active modulators of senescence-associated signaling rather than passive lipid stores, this review highlights how disruption of senescence–lipid feedback loops may represent a disease-modifying opportunity in MASLD progression.

## 1. Introduction

Metabolic dysfunction-associated steatotic liver disease (MASLD), previously referred to as non-alcoholic fatty liver disease (NAFLD), is a prevalent chronic liver disorder characterized by abnormal lipid accumulation within lipid droplets (LDs) in over 5% of hepatocytes [1]. The global prevalence of MASLD has increased markedly, affecting more than one-third of the adult population worldwide. The burden is particularly high in Western populations, as MASLD is closely associated with type 2 diabetes (T2D) and obesity [2].

In approximately 15% of patients, MASLD progresses to metabolic dysfunction-associated steatohepatitis (MASH), and among these, up to 30% may develop fibrosis, which can further progress to cirrhosis and, ultimately, hepatocellular carcinoma (HCC) [3].

Several studies have highlighted the presence of senescent hepatocytes during the development of MASLD, suggesting that dysregulated cellular senescence (CS) may play a key role in its pathogenesis [4,5]. A chronically diseased liver generates an enormous burden of CS, as up to 80% of hepatocytes have been shown to become senescent in advanced stages [4,6,7]. Changes in the metabolic activity of senescent hepatocytes, particularly in lipid regulation, have been shown to further exacerbate steatotic conditions [8]. Liver function deterioration has also been associated with prolonged CS [6]. However, the causal relationships between CS, lipid dysregulation, and MASLD progression are far from being clearly defined.

Unlike previous reviews that primarily discuss CS or hepatic lipid metabolism as largely independent or downstream processes in MASLD, the present review specifically addresses their bidirectional and stage-dependent crosstalk. By focusing on LDs not only as passive lipid-storage organelles but also as active modulators of senescence-associated metabolic and inflammatory signaling, this review provides an integrative framework for understanding how CS–LD interactions contribute to disease progression from simple steatosis to MASH.

## 2. CS

### 2.1. Stages and Biomarkers

CS, which denotes stable cell-cycle arrest, is a complex, multistep biological process that is often divided into three stages: early, full, and late senescence [9]. CS can also be classified into two main types: replicative senescence (RS) and stress-induced premature senescence (SIPS). RS is primarily driven by telomere shortening and occurs during normal aging, affecting all somatic cells capable of proliferation. In contrast, SIPS is mainly telomere-independent and is triggered by various internal or external stressors, including mitochondrial dysfunction, oxidative stress, DNA damage (mainly induced by chemotherapeutic drugs, also referred to as treatment-induced senescence, TIS), and oncogene activation [10]. Nevertheless, RS and SIPS depend on similar pathways, as it is well-known that the p53-p21 and p16INK4α-Rb signaling cascades are activated during the early stages of the CS program [11].

Since there is no single biomarker specific to CS, molecules involved in these pathways, along with reduced expression of proliferation markers such as Ki-67, act as important indicators of CS [12]. Senescence-associated beta-galactosidase (SA-β-gal) is widely considered the gold standard for detecting senescent cells. However, recent data have raised concerns about its specificity, as a rapid increase in SA-β-gal levels is not exclusive to the CS state. Instead, it has also been observed in senescent human mesenchymal stromal cells that either recover from stressful stimuli and successfully reenter the cell cycle or, alternatively, enter into the CS state [12]. Furthermore, it was found to be constitutively expressed in several tissues, including neurons [13]. Lipofuscin, a yellowish-brown pigment referred to as the “age pigment”, is also considered an established biomarker for CS [14]. Lipofuscin is accumulated in senescent cells as a result of lysosomal dysfunction and is composed of incompletely degraded molecules, such as lipids, proteins, and sugars [15]. However, lipofuscin is not detected only in senescent cells; it has also been found in metabolically active postmitotic cells, such as cardiomyocytes and certain neurons that retain proliferative capacity [16]. SA-β-Gal and lipofuscin were found to co-localize, suggesting that these markers could be used interchangeably to detect CS [17]. Epigenetic changes such as senescence-associated heterochromatin foci (SAHF), regions of heterochromatin that suppress proliferation-promoting genes in senescent cells [18], and senescence-associated DNA damage foci (SADF), which contain key proteins for DNA-damage response (DDR) and CS, also serve as potential CS biomarkers [19].

### 2.2. Senescence-Associated Secretory Phenotype

CS is accompanied by the senescence-associated secretory phenotype (SASP), which includes various proteins, cytokines, chemokines, growth factors, and proteases [20]. SASP is highly complex, dynamic, and heterogeneous, varying across different cell types [21], senescence-inducing stimuli [22], and stages of CS [23]. It is regarded as the most influential characteristic of senescent cells, enabling them to communicate with their surrounding microenvironment through paracrine actions [24]. At the same time, in an autocrine manner, SASP factors reinforce the CS program within cells.

SASP secretion can be divided into three main phases: an initial rapid phase associated with the DNA damage response (DDR) pathway, followed by a slower self-amplifying phase, and ultimately a late or “mature” SASP phase [25]. SASP involves modifications to both soluble factors and extracellular vesicles (EVs). Soluble SASP factors include interleukin (IL)-1, IL-6, IL-8, chemokines such as Monocyte Chemoattractant Protein-1 (MCP-1), growth factors such as Hepatocyte Growth Factor (HGF), and proteases such as Matrix Metalloproteinases (MMPs) [26]. Additionally, data from senescent human dermal fibroblasts indicate that cellular components, such as proteins and lipids contained in EVs, also act as SASP factors by facilitating intercellular communication [27]. Senescent cells release a large amount of EVs, and the induction of CS results in changes in the lipid composition of EVs [28].

Evidence from in vitro studies of human fibroblasts indicates that early SASP features emerge within a few days of SIPS induction. During this phase, inflammatory SASP-associated factors, such as IL-1, can be detected at very low levels in the extracellular milieu [20]. Based on experimental studies, early senescent cells appear to secrete these factors as part of an adaptive response that supports tissue repair and wound healing, highlighting the beneficial effects of the transient presence of senescent cells [29]. Accumulating evidence indicates that the SASP is highly dynamic, with early senescence marked by significant heterogeneity in secretory profiles, while later or stable senescent states display greater similarity in SASP composition, reflecting the development of a conserved core secretome [29]. This early heterogeneity likely reflects the transitional nature of CS and its dependence on initial cellular conditions and stressors. As senescence stabilizes, common functional adaptations, such as metabolic reprogramming, ECM remodeling, and immune modulation, tend to converge, resulting in a more conserved senescent phenotype [29,30]. The early SASP phase also marks the initiation of slow, self-amplifying autocrine loops that gradually intensify the production and secretion of SASP factors [20].

As cells progress to full senescence, they undergo extensive chromatin remodeling, which is largely attributed to the reduction in lamin B1, a structural protein of the nuclear membrane [31]. This decrease in lamin B1 leads to a “leaky” nucleus, promoting shifts in SASP gene expression [32]. During this stage, SASP alterations primarily involve enrichment of pro-inflammatory factors through positive feedback loops that promote age-related diseases, such as MASLD [29]. Nuclear factor-kappa B (NF-κΒ) is also implicated in the regulation of key inflammatory SASP components, such as IL-1, IL-6, and IL-8, and these cytokines act within a positive feedback loop to further promote NF-κB expression and enhance SASP signaling [33]. It was recently described that sustained NF-κB activity enhanced inflammatory gene expression through increased NF-κB-DNA binding and slowed the cell cycle [34]. Another regulator of SASP is mTOR, which inhibits SASP-mRNA-degrading proteins, thus stabilizing SASP expression and reinforcing CS [35]. Following continuous self-amplification, SASP eventually reaches a mature state, which is the hallmark of late-senescent cells [25]. Further upregulation of p16 is implicated in maintaining the irreversible nature of the senescence state, increasing SA β-gal activity, and altering mitochondrial metabolism [36].

Apart from nuclear changes, senescent cells also display prominent cytosolic characteristics. A widely recognized hallmark of CS is the enlarged cellular morphology accompanied by an increased number and mass of cellular organelles, such as lysosomes and mitochondria, as well as alteration in plasma membrane composition [37]. Cell enlargement during CS is largely attributed to the activation of the mammalian target of rapamycin (mTOR) pathway [38], facilitating processes such as nutrient uptake and synthesis of proteins and lipids, both contributing to the enhanced cell size [39]. Additionally, the increased mass of lysosomes has been linked to the accumulation of the lysosomal enzyme SA-β-gal [40,41]. Finally, senescent cells may either undergo immune-mediated clearance or persist in a more profound senescent state.

In this late phase, various pro-inflammatory factors are upregulated, including cytokines (e.g., IL-6 and IL-8), ECM-remodeling proteins, immune-evasion molecules, and factors involved in stress responses and metabolic regulation [29]. IL-1 has been associated with the paracrine activity of senescent cells, as it can induce normal adjacent cells to enter the CS program [25,42,43]. Interestingly, specific microRNAs, such as miR-146a and miR-146b, can regulate SASP secretion to prevent excessive amplification [25]. If this modulation fails to attenuate SASP secretion, a more stable, long-term SASP signature is established [25]. This phase is also described as the stable late SASP phase and typically occurs approximately 30 days or more after the persistence of senescent cells. During this stage, pro-inflammatory cytokines dominate, contributing to the detrimental effects of CS, including chronic inflammation, tumor oncogenesis, and aging [29]. However, despite significant advances in characterizing SASP dynamics, only a limited number of studies have investigated its regulation, and most mechanistic insights are derived from non-hepatic in vitro systems or animal models, with limited direct evidence in human metabolic liver disease.

### 2.3. Lipogenesis in Senescent Cells

Considering the above-mentioned features of CS, such as membrane remodeling, increased cellular mass, and SASP secretion, especially through the lipid-enriched EVs, lipids are considered key modulators for the establishment and maintenance of CS [44]. Evidence from lipidomics and mechanistic analyses indicates that CS is accompanied by a broad rewiring of lipid-related pathways, including alterations in lipid uptake, biosynthesis, and fatty acid oxidation (FAO), which contribute to the distinct metabolic phenotype of senescent cells and the accumulation of LDs [44].

Lipid intake capacity [45] represents a feature possibly involved in preserving the senescent phenotype, including membrane remodeling and SASP [46]. Senescent cells, mainly in lipid-rich tissues such as hepatic and adipose tissue [47], are characterized by increased levels of cluster of differentiation (CD) 36, a membrane receptor involved in fatty acid uptake and lipid metabolism. CD36 is also implicated in the induction of CS of adjacent cells and in the modulation of SASP [48]. Notably, the initial upregulation of CD36, observed rapidly upon CS, is required for the activation of crucial transcription factors, including NF-κB, that drive SASP formation [46]. Importantly, the sustained expression of CD36 promotes the secretion of pro-inflammatory SASP components, such as IL-6 and IL-8, thereby contributing to the establishment of a full senescent state [48].

Lipid biosynthesis is also activated during the initial stages of CS, as the increased cellular mass and accumulation of membranous organelles, such as mitochondria and lysosomes in senescent cells, are highly dependent on lipid biosynthesis [37]. This upregulation relies on key rate-limiting enzymes, such as fatty-acid synthase (FASN), which has been shown to be elevated before late-stage senescence begins [49]. Besides membranous remodeling, studies have revealed that reducing FASN levels inhibits SASP, emphasizing the vital role of lipid biosynthesis in developing the secretory phenotype of early senescent cells [49].

LDs are highly dynamic organelles that play a central role in lipid metabolism, storage, and cellular signaling. They are composed of a core of neutral lipids, primarily triglycerides (TGs) and cholesteryl esters, surrounded by a phospholipid monolayer embedded with proteins, such as perilipins (PLINs), that regulate lipid metabolism and LDs’ interactions with other organelles. Thus, LDs mitigate lipotoxicity by sequestering excess fatty acids as TGs [50]. They are also recognized as significant regulators of cellular homeostasis as they interact with various organelles, including mitochondria, endoplasmic reticulum, and peroxisomes, to regulate lipid flux and energy balance [51]. Beyond their metabolic functions, LDs can also modulate intracellular signaling pathways, immune responses, and membrane trafficking [52], and disruption of their homeostasis can lead not only to dysregulation of lipid metabolism but also to alterations in cellular signaling pathways [53]. Abnormalities in lipid metabolism can further lead to hepatic steatosis, inflammation, and oxidative stress [21].

### 2.4. Mitochondrial Dysfunction in Senescent Cells

Accumulating evidence indicates that mitochondria are not only passive responders to metabolic rewiring during CS but also active regulators of its initiation, maintenance, and propagation [54]. During the initial phase of CS, mitochondria undergo rapid and coordinated remodeling events that reflect both structural and functional adaptations. Μechanistic insights derived from in vitro senescence models indicate that early stages of CS are accompanied by pronounced mitochondrial structural remodeling. In particular, CS has been associated with the formation of elongated and enlarged mitochondria due to an imbalance in fusion and fission processes [22]. This reorganization is not merely structural; it directly contributes to a transient enhancement of FAO, allowing the cell to generate additional pools of acetyl-CoA that are required for metabolic adaptation and epigenetic remodeling, such as increased histone acetylation. Consequently, these metabolic alterations contribute to further enhancement of p16 expression, thereby reinforcing cell-cycle arrest [23]. In this context, proteins associated with the DDR, particularly ataxia-telangiectasia mutated (ATM) kinase, play a central role. ATM activation not only propagates canonical DNA repair signals but also transduces stress cues to mitochondria, thereby inducing CS through upregulation of FAO pathways [23]. Furthermore, activation of p53 during the early phase of CS results in upregulation of key enzymes implicated in mitochondrial FAO [55].

As CS progresses toward a more stable and irreversible state, mitochondrial physiology changes dramatically. Mitochondria frequently become enlarged, elongated, and functionally compromised, with impaired oxidative phosphorylation, declining ATP output, and excessive generation of ROS [56]. Consequently, studies of late senescent hepatocytes have shown impaired mitochondrial FAO, leading to lipid accumulation and metabolic inflexibility [57]. Interestingly, ROS have been shown to promote the activation of pro-inflammatory components of SASP, mainly via NF-κΒ activation, creating a feed-forward inflammatory loop that reinforces the senescent state [58].

## 3. MASLD Pathogenesis

### 3.1. Microvesicular and Macrovesicular Types of Steatosis

Nascent LDs originate from the outer membrane of the ER, where neutral lipids accumulate [26]. Under normal conditions, the size of LDs is tightly regulated, and hepatocytes primarily contain smaller LDs, which are more metabolically active, allowing for efficient interaction with lipases and cytosolic organelles, such as mitochondria. However, in MASLD, this balance is disrupted. Hepatocytes, in an attempt to compensate for the elevated influx of free fatty acids (FFAs), sequester them as TGs within LDs, a process known as steatosis [59]. The type of steatosis observed in MASLD is also influenced by LD structure; earlier stages of MASLD are typically characterized by macrovesicular steatosis, which entails a large droplet of fat within the hepatocyte, displacing the nucleus to the periphery of the cytoplasm. The presence of macrovesicular steatosis is mainly associated with a favorable long-term prognosis, as these large LDs serve as long-term lipid reservoirs, thus protecting the liver against lipotoxicity [60]. However, as MASLD progresses to more severe stages, such as MASH, hepatocytes display a coexistence of macrovesicular steatosis along with aggregates (“patches”) of microvesicular steatosis, which are described as enlarged hepatocytes with a foamy, vacuolated cytoplasm, known as balloon cells [60]. Microvesicular steatosis has been associated with more severe grades of macrovesicular steatosis, inflammation, oxidative stress, and mitochondrial dysfunction [61]. These LD aggregates may indicate regions of hepatocytes under increased metabolic stress, where defective FAO leads to the formation of amphiphilic compounds that contribute to the development of microvesicular steatosis [62]. The coexistence of both forms of steatosis suggests a spectrum of hepatocellular injury, where different hepatocytes or liver regions undergo varying degrees of lipid dysregulation.

### 3.2. The Implication of PLINs

Apart from changes in size, the protein composition of the LD surface is altered during MASLD, playing a key role in lipid metabolism and thereby contributing to disease progression. Notably, PLINs, the most abundant LD-associated proteins, are strongly associated with MASLD progression [63].

The predominance of PLIN2 in the steatotic liver suggests that the balance between lipid storage and mobilization is severely disrupted, favoring lipid retention over lipid utilization [64]. This imbalance not only exacerbates steatosis but also predisposes hepatocytes to additional metabolic stress, contributing to the transition from simple steatosis to more advanced stages of liver disease, including metabolic-associated steatohepatitis (MASH) and fibrosis. PLIN2 was found to be the most upregulated PLIN in the steatotic liver, being responsible for LD formation by promoting the sequestration of TGs within LDs and inhibiting the access of lipolytic mechanisms to LDs [65,66], leading to excessive LD accumulation and persistence of steatosis [67]. Moreover, PLIN2 levels were also related to mitochondrial dysfunction, leading to the induction of CS through the upregulation of stress-related proteins [68].

PLIN3 has been found to be upregulated in human steatotic livers [69], and its silencing has been shown to reduce steatosis [26]. PLIN5, typically expressed in oxidative tissues such as liver and muscle, is also upregulated in MASLD [70]. When cells are overloaded with lipids, PLIN5 inhibits the recruitment of cytosolic lipases to LDs and mitochondrial β-oxidation, thus protecting the liver against lipotoxicity [71]. Moreover, PLIN5 has been implicated in modulating inflammatory responses, as its deficiency has been shown to increase oxidative stress, which may, in turn, activate pro-inflammatory pathways, including NF-κB signaling [72].

### 3.3. PPARa and NF-κΒ

The complex interplay between impaired lipid metabolism and chronic inflammation primarily drives the progression of MASLD from simple steatosis to severe liver damage. Among the key regulatory pathways, PPARα, which controls lipid metabolism, and NF-κΒ, a key regulator of inflammation, play crucial roles in the development of the disease.

PPARα is a nuclear receptor that regulates FAO and lipid homeostasis, helping to mitigate lipid accumulation [73]. It is a well-established modulator of MASLD pathogenesis through its complex interaction with mitochondria and inflammatory signaling pathways [73]. Under normal conditions, PPARα activation facilitates mitochondrial biogenesis and function, ensuring efficient FAO and preventing excess lipid accumulation [73]. In early MASLD, PPARα serves as a compensatory mechanism, counteracting lipid overload by promoting FAO [74]. PPARα activation suppresses NF-κB-dependent transcription of cytokines, such as IL-6, IL-1β, and TNF-α, thereby limiting hepatic inflammation [75]. However, as MASLD progresses, PPARα expression declines, leading to impaired mitochondrial function, reduced ATP production, and increased oxidative stress [76]. This mitochondrial dysfunction exacerbates lipid accumulation, contributing to hepatocellular damage and inflammation. Additionally, PPARα downregulation disrupts LD metabolism, resulting in impaired lipolysis and excessive lipid accumulation within hepatocytes [73]. This lipid overload may induce lipotoxicity and promote inflammatory signaling pathways, which further accelerate MASLD progression.

In contrast, NF-κB is a central mediator of inflammatory responses, since its sustained activation drives the secretion of pro-inflammatory cytokines through SASP, contributing to persistent inflammation, hepatocyte injury, and fibrosis progression [75], representing a key player in the progression of steatosis to MASH. Under normal conditions, NF-κB activity is strictly regulated, ensuring that inflammatory pathways are activated in response to a stressful stimulus, while preventing persistent inflammation once the stimulus has been resolved [77]. This tight regulation prevents chronic inflammation and uncontrolled tissue damage in the liver, maintaining hepatic homeostasis. However, in MASLD, chronic metabolic stress, lipotoxicity, and oxidative damage trigger persistent NF-κB activation, leading to sustained inflammation and hepatocyte injury [78]. Activated NF-κB induces the transcription of pro-inflammatory cytokines, including IL-6, IL-1β, and TNF-α, which perpetuate hepatic inflammation and immune cell recruitment [79]. Additionally, NF-κB interacts with the SASP, amplifying the release of inflammatory mediators that exacerbate hepatocellular stress and fibrosis [25].

The crosstalk between PPARα and NF-κB determines whether the liver adapts to metabolic stress or succumbs to progressive damage. In the early stages of MASLD, elevated levels of PPARα play a protective role against lipid overload through the activation of FAO [74]. Moreover, PPARα activation has a direct anti-inflammatory effect by suppressing NF-κB-dependent transcription of key cytokines, which are central to the inflammatory response in the liver [75]. Conversely, the downregulation of PPARα in advanced stages of MASLD contributes significantly to the propagation of the disease by promoting excessive activation of NF-κB, resulting in chronic inflammation, hepatocellular senescence, and fibrosis [75]. NF-κB-mediated inflammation enhances the production of SASP factors, which perpetuate a pro-inflammatory microenvironment, thereby accelerating disease progression [33]. Studies have shown that oxidative stress is an important mechanism of NF-κB activation in the liver [80], and it is also associated with DNA damage and genomic instability, thus contributing to hepatocellular dysfunction and disease progression.

### 3.4. Mitochondrial Dysregulation

Mitochondrial dysfunction, observed by altered mitochondrial morphology and decreased oxidative capacity, represents a well-established driver of hepatοcellular steatosis [81,82] and is considered one of the main hallmarks of MASLD, especially during MASH [83]. Elevated fatty-acid influx towards the liver results in an initial upregulation of mitochondrial fatty-acid oxidation, which acts as a compensatory mechanism to prevent lipid accumulation, a process known as “mitochondrial flexibility” [82]. However, this adaptation in mitochondrial metabolism does not persist in more severe stages like MASH, during which hepatocytes display decreased energy production and impaired mitochondrial respiration [81]. However, prolonged elongated mitochondria ultimately result in higher production of reactive oxygen species (ROS) and decreased mitochondrial respiration activity [56]. Interestingly, ROS was found to promote the activation of pro-inflammatory components of SASP [58]. Moreover, prolonged FAO results in epigenetic alterations, such as overexpression of p16 [23]. In a recent study, overexpression of p16 in primary hepatocytes was shown to reduce mitochondrial activity and FAO and led to accumulation of LDs both in vitro and in vivo [84], pointing out a potential vicious cycle between CS and mitochondrial metabolism.

## 4. Interplay Between CS and LDs in MASLD

**Figure 1:**
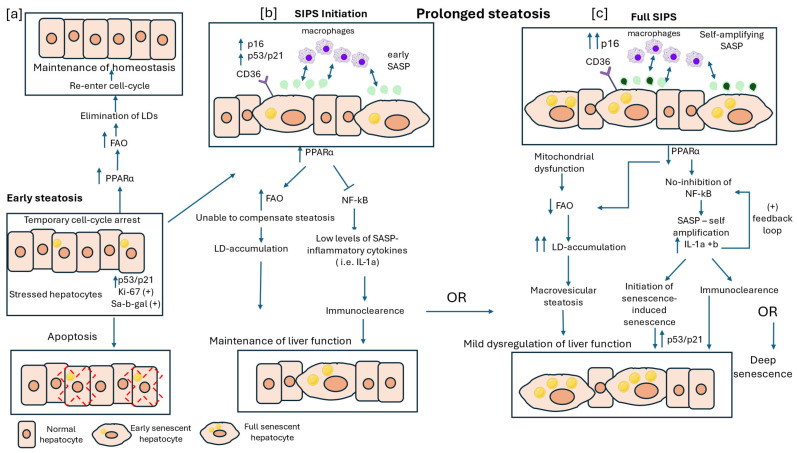
Hepatocyte Senescence and Lipid Dysregulation in the Early Stages of Metabolic Dysfunction-Associated Steatotic Liver Disease (MASLD). This figure illustrates the interaction between stress-induced premature senescence (SIPS) and lipid droplets (LDs) in early stages of MASLD. (**a**) Early steatosis is characterized by transient cell-cycle arrest, with stressed hepatocytes expressing p53/p21, Ki-67, and senescence-associated β-galactosidase (SA-β-gal). These hepatocytes may either undergo apoptosis or recover through fatty acid oxidation (FAO) activation, mediated by increased levels of peroxisome proliferator-activated receptor alpha (PPARα). (**b**) In cases of prolonged steatosis, hepatocytes undergo SIPS initiation, marked by upregulation of p16, p53/p21, and early senescence-associated secretory phenotype (SASP) cytokines such as IL-1α, which can recruit macrophages for immune-mediated clearance of senescent hepatocytes. However, the increased levels of PPARα are unable to compensate for excess fatty acid influx and, along with increased levels of CD36, a lipid-associated receptor, lead to lipid accumulation. If steatosis persists and immune clearance of senescent hepatocytes is unsuccessful, these cells enter full SIPS. (**c**) During this phase, PPARα levels are downregulated, leading to reduced FAO, while early mitochondrial dysregulation contributes to further lipid accumulation, known as macrovesicular steatosis. This phase is also characterized by self-amplifying SASP, persistent nuclear factor kappa B (NF-κB) activation due to reduced PPARα expression, and a positive feedback loop of SASP pro-inflammatory cytokines. Additionally, SASP factors exert paracrine effects, triggering senescence in adjacent normal hepatocytes. Depending on the efficiency of immune clearance, hepatocytes in full SIPS may either be eliminated, allowing partial restoration of liver function, or persist, leading to deep senescence and progressive liver dysfunction.

At the cellular level, studies across different experimental systems indicate that CS can develop gradually in response to metabolic stressors, such as increased FFA influx, in competition with alternative cell-fate programs, including transient cell-cycle arrest, cell death, and proliferation, resulting in a heterogeneous cell population [45]. Supporting this concept, cell-based studies in human mesenchymal stromal cells and fibroblasts have demonstrated that stressed cells may display a pre-senescent phenotype, characterized by the concomitant expression of proliferation-associated markers (e.g., Ki-67) and senescence-associated features, such as SA-β-gal activity [12]. When the stressful insult is time-limited, attenuation of cellular stress may allow cells to escape CS and potentially reenter the cell cycle [3] (Figure 1a). In contrast, persistent stress promotes stabilization of the senescent program, marked by sustained upregulation of p16, in addition to p53/p21 signaling, which ensures the irreversibility of CS [85]. Evidence from in vitro cellular models indicates that induction of CS is accompanied by enhanced lipid uptake, as reflected by increased expression of CD36 [48] and lipid biosynthesis [37], as pointed by elevated levels of FASN [49]. However, increased FFA influx can simultaneously activate PPARα, thereby enhancing FAO and acting as an efficient compensatory mechanism limiting steatosis during early disease stages [74].

Beyond its role in lipid uptake, experimental in vitro studies have shown CD36 upregulation during CS can also drive aspects of the SASP through NF-κB–dependent signaling, highlighting CD36 as a molecular link between lipid signaling and secretory phenotype activation [48]. SASP represents a central hallmark of CS and a crucial regulator of MASLD pathogenesis [86]. Interestingly, early SASP involves mainly growth factors and cytokines such as IL-1α and β, which are initially secreted at low levels [20,24]. In early MASLD, PPARα activation may attenuate NF-κB signaling, thereby limiting excessive SASP-mediated inflammation [75]. At the same time, low levels of secreted SASP cytokines can induce immune-mediated clearance of scattered senescent hepatocytes, thereby preserving liver regenerative capacity and normal hepatic function (Figure 1b).

As steatosis persists, hepatocytes progress to a late senescent state. Of note, increased mitochondrial mass and subsequent increased FA oxidation are associated with significant transcriptional changes, such as epigenetic induction of p16 expression, thus maintaining permanent cell-cycle arrest and promoting CS reprogramming [23,36]. In contrast, overexpression of p16 results in mitochondrial deregulation and a gradual decrease in β-oxidation capacity [84]. This metabolic reprogramming plays a protective role since cells are able to sequester FAs, which are highly reactive species prone to oxidation, and store them away from membranes, thus limiting membrane damage under oxidative conditions [44]. Prolonged enhanced influx of FFAs, combined with p16-induced initial decline in mitochondrial function, may contribute to the significant LD accumulation seen during MASLD progression. However, further investigations comparing LD accumulation between senescent and proliferative steatotic hepatocytes are necessary to fully understand the interaction between these two cell populations and determine whether senescent hepatocytes accumulate more LDs than their proliferative counterparts. In addition to LDs, senescent steatotic hepatocytes secrete SASP factors, entering a self-amplifying phase characterized by significantly increased levels of IL-1α and IL-1β [20]. These cytokines act as positive feedback signals, stimulating the production of additional pro-inflammatory cytokines, such as IL-6 and IL-8. Moreover, as MASLD progresses, levels of PPARα begin to decrease, contributing further to mitochondrial downregulation and activation of NF-κB, which promotes the secretion of pro-inflammatory cytokines (Figure 1c).

Importantly, accumulating evidence indicates that CS in non-parenchymal liver-resident cell types actively contributes to MASLD progression, rather than representing a passive consequence of hepatocyte dysfunction. Kupffer cells and monocyte-derived macrophages acquire senescence-associated features under chronic metabolic and oxidative stress, contributing to sustained SASP production [80]. Notably, when hepatic macrophages are cultured in medium from senescent HepG2 cells, they tend to migrate as inflammatory-type macrophages [87] and contribute to the inflammatory milieu of MASH. Under these inflammatory conditions, hepatic stellate cells (HSCs) become activated, promoting fibrogenesis and establishing feed-forward inflammatory loops that drive MASLD progression [80]. However, HSCs also undergo senescence in response to chronic liver injury. Studies in experimental models have shown that while transient HSC senescence may initially exert antifibrotic effects by limiting proliferation, persistent senescent HSCs contribute to fibrosis progression through SASP-mediated extracellular matrix remodeling and profibrogenic signaling [88]. Liver sinusoidal endothelial cells (LSECs) represent another critical senescent cell population in MASLD. Senescent LSECs acquire a pro-inflammatory secretory phenotype, which disrupts sinusoidal homeostasis and facilitates immune cell recruitment [89]. Through SASP-mediated crosstalk with hepatocytes, macrophages, and HSCs, senescent LSECs further amplify inflammation and tissue remodeling in MASLD [90].

## 5. Interaction Between SIPS and RS During the MASH Progression

Senescent hepatocytes have been shown to increase in number as chronic liver disease advances, highlighting their potential role in disease progression [91]. Persistent liver insults, such as steatosis, drive the continuous production of senescent hepatocytes, leading to their accumulation, a process known as chronic senescence [3]. Notably, senescence biomarkers, such as p21, correlate positively with the MASLD activity score [92], with further increases in more severe stages, such as MASH [6]. This increase in p21 may be attributed to the progressive induction of CS in more and more neighboring hepatocytes, contributing to the damage of hepatic architecture and the deterioration of liver function.

**Figure 2:**
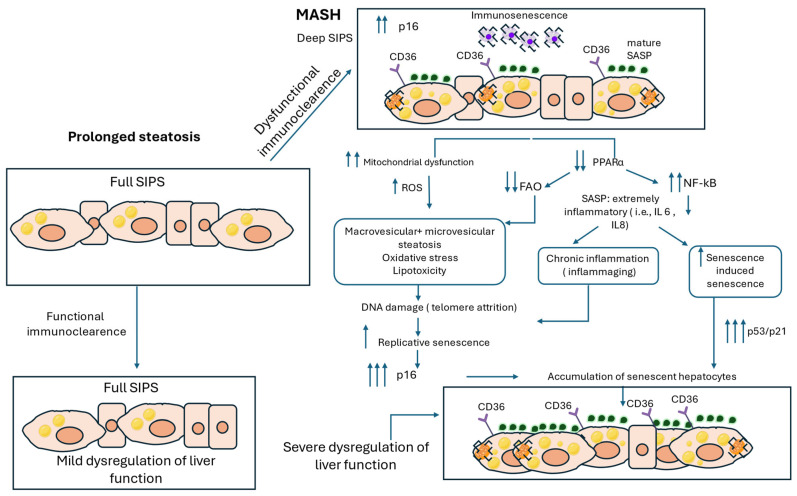
Hepatocyte Senescence and lipid dysregulation in metabolic dysfunction-associated steatohepatitis (MASH) Progression. This figure illustrates the molecular mechanisms linking the interaction of stress-induced premature senescence (SIPS) and lipid droplets (LDs) to MASH progression. In deep SIPS, hepatocytes exhibit sustained upregulation of p16 and mature senescence-associated secretory phenotype (SASP) factors, promoting a chronic pro-inflammatory environment. Increased CD36 expression further exacerbates lipid accumulation, while immune senescence impairs the clearance of senescent hepatocytes. Mitochondrial dysfunction is another hallmark of deep senescent cells, leading to increased reactive oxygen species (ROS) production. Sustained mitochondrial impairment, along with extremely downregulated levels of peroxisome proliferator-activated receptor alpha (PPARα) and fatty-acid oxidation (FAO), results in both macrovesicular and microvesicular steatosis, oxidative stress, and lipotoxicity, all of which contribute to chronic liver inflammation. Additionally, amplification of nuclear factor kappa B (NF-κB) signaling enhances SASP-driven secretion of highly inflammatory cytokines, such as IL-6 and IL-8, which in turn promote senescence in neighboring hepatocytes. Persistent oxidative stress and lipid toxicity drive DNA damage, which disproportionately affects telomeres, leading to telomere attrition and triggering replicative senescence marked by p53/p21 upregulation. The accumulation of senescent hepatocytes, combined with ineffective immune-mediated clearance, contributes to progressive liver dysfunction and MASH progression.

During later stages of SIPS, common functional changes emerge, such as metabolic rewiring to support the survival of senescent cells, modifications in the extracellular matrix (ECM), and immune system modulation [29]. Concurrently, PPARα levels are further decreased during MASH, thus exacerbating mitochondrial beta-oxidation impairment in senescent hepatocytes [93]. This downregulation of PPARα also elevates NF-κB activity, leading to a sustained production of pro-inflammatory SASP factors, such as IL-1, IL-6, and TGF-β, which in turn promote CS in adjacent cells [94]. This process, referred to as senescence-induced senescence, contributes to the clustering of senescent hepatocytes [95]. The prolonged exposure to pro-inflammatory SASP factors not only sustains the senescent phenotype, but also drives the transition into its ‘mature’ state following continuous self-amplifying loops [25] (Figure 2).

Beyond the well-established role of SASP in the pathogenesis of MASH, metabolic dysregulation, such as impaired mitochondrial function, may also play a crucial role in the propagation of the disease [96]. Mitochondrial dysfunction in MASH has been associated with microvesicular steatosis [97]. The coexistence of microvesicular and macrovesicular steatosis is a well-known histological feature of MASH livers [61], which may be explained by impaired mitochondrial function and dysregulated lipid metabolism. More specifically, it is plausible that the increasing uptake of fatty acids exceeds the capacity of senescent cells to store them in large LDs. The residual non-esterified FAs, which cannot be stored in LDs, may form amphiphilic compounds in the cytoplasm, shown as fat deposits [62]. Together with severe mitochondrial dysfunction, this results in the development of patches of microvesicular steatosis, near ballooned hepatocytes [60], regions associated with increased inflammation and oxidative stress.

Considering that MASLD is characterized by prolonged oxidative stress and thus increased DNA damage, it is plausible that the accumulation of senescent hepatocytes and the persistent “mature” SASP signaling further contribute to telomere attrition and genomic instability. This, in turn, could amplify RS during MASH. Thus, it is proposed that both SIPS and RS are implicated in the propagation of CS [3], highlighting the multifaceted role of hepatocyte senescence in MASLD progression. Consistent with this notion, studies have shown that telomere attrition, along with DNA damage, is more pronounced in hepatocytes at more severe stages of steatosis [6], possibly indicating the induction of both RS and SIPS in more chronic steatotic conditions. Both mechanisms share similarities, including activation of the p53 pathway triggered by DNA damage and critical telomere shortening [98]. The percentage of hepatocytes with DNA damage increased in parallel with steatosis grade, and concomitantly, hepatocytes with DNA damage had significantly shorter telomeres, apparently because DNA damage disproportionately affects telomere length due to less efficient repair mechanisms [99]. Furthermore, chronic LD accumulation and subsequent persistent oxidative stress may induce both DNA damage and telomere shortening in hepatocytes [6]. These observations suggest that the initial activation of SIPS by LD accumulation may progressively enhance RS, leading to a marked accumulation of senescent cells, particularly in aged patients who already harbor an increased senescent cell burden [100]. In line with this, premature telomere shortening has been detected in hepatocytes in MASLD [6], further supporting the notion that persistent liver injury may amplify RS and highlighting a potential interplay between SIPS and RS during MASLD progression.

## 6. Therapeutic Perspectives Linking CS Modulation and LD Metabolism in MASLD

The bidirectional interplay between CS and LD metabolism provides multiple therapeutic entry points for mitigating MASLD. Current strategies targeting the CS–LD axis can be broadly categorized into three approaches: (i) elimination or functional reprogramming of senescent cells, (ii) restoration of lipid metabolic homeostasis, and (iii) preservation of mitochondrial integrity alongside suppression of SASP-driven inflammation.

Senolytic agents, including dasatinib combined with quercetin, navitoclax, and fisetin, selectively eliminate senescent hepatocytes by disrupting pro-survival signaling pathways. Preclinical evidence from experimental models of MASLD indicates that treatment with dasatinib plus quercetin reduces the burden of senescent cells, attenuates hepatic lipid accumulation, and suppresses the expression of profibrotic genes, such as TGF-β1, thereby improving histopathological features of disease [101]. However, accumulating data suggest that senolytic efficacy is highly stage-dependent. In MASLD-associated HCC mouse models, early senolytic intervention during the steatosis phase failed to reduce senescence, whereas delayed administration during the MASH phase markedly decreased senescent hepatocyte burden [102]. These observations underscore a critical limitation of senolytic strategies, namely the potential disruption of transient and possibly adaptive senescence responses when such interventions are applied prematurely.

In contrast to senolytics, senomorphic therapies aim to modulate the phenotype and secretory activity of senescent cells, without inducing cell death, primarily by attenuating the SASP [103]. Rapamycin, one of the most extensively characterized senomorphic agents, suppresses SASP signaling by inhibiting the mechanistic target of the mTOR pathway. In murine models of hepatic CS, rapamycin markedly reduced SASP expression and immune cell infiltration [35]. Similarly, metformin, a widely prescribed antidiabetic drug, attenuates SASP production by activating AMPK and inhibiting NF-κB-dependent inflammatory signaling [104].

Beyond direct senescence-targeting strategies, restoring lipid metabolic homeostasis is an essential therapeutic pillar. PPARα has emerged as a promising therapeutic target due to its central role in regulating FAO, lipid homeostasis, and inflammation. A novel class of agents, selective PPARα Modulators (SPPARMα), such as pemafibrate, demonstrates greater selectivity and efficacy in enhancing hepatic β-oxidation and reducing steatosis [105]. Clinical evidence suggests that PPARα activation can improve liver function and may synergize with anti-inflammatory approaches, broadening the therapeutic landscape for MASLD [106]. Additionally, a recent multicenter, randomized, open-label clinical study of pemafibrate in adult patients with MASLD/MASH provides human-based evidence of improved metabolic and hepatic parameters following PPARα activation [107]. However, the non-histological endpoints of the study limit conclusions regarding direct effects on hepatocellular senescence burden across the disease stages.

Finally, emerging agents that directly target hepatic lipid metabolism may be rationally combined with senescence-focused interventions. Resmetirom, a thyroid hormone receptor β agonist, is the first FDA-approved treatment for MASH and has been shown to significantly reduce hepatic fat content and to improve noninvasive markers of fibrosis [108]. Given the documented crosstalk between thyroid hormone signaling and CS-related pathways, including SIRT1- and FOXO-mediated stress responses, combination strategies integrating metabolic modulators with senescence-targeted therapies warrant systematic investigation in future translational and clinical studies.

## 7. Conclusions

MASLD progression is increasingly recognized as a process tightly linked to the accumulation and persistence of senescent hepatocytes. Beyond serving as passive markers of disease severity, senescent cells actively reshape hepatic metabolism through coordinated alterations in lipid handling, mitochondrial function, and inflammatory signaling. Framing CS and lipid metabolism as a coupled, stage-dependent pathogenic axis highlights the potential of therapeutically targeting senescence–lipid feedback loops, particularly including CS induction and SASP modulation, as disease-modifying strategies in MASLD.

**Figure 3:**
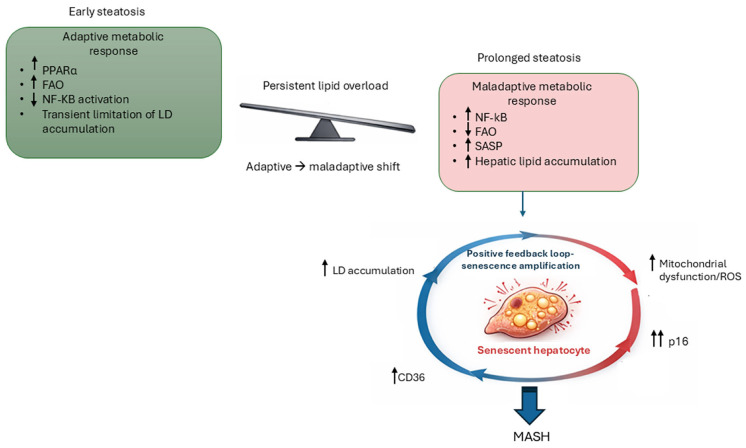
Mechanistic integration of cellular senescence and lipid droplet dysregulation in MASLD. This figure illustrates a stage-dependent model linking hepatic lipid overload to the induction of cellular senescence (CS) and accumulation of lipid droplets (LDs). During early steatosis, increased free fatty acid (FFA) influx activates a peroxisome proliferator-activated receptor alpha (PPARα)-dependent metabolic program that enhances fatty acid oxidation (FAO) and suppresses NF-κB signaling, constituting a transient adaptive response. Persistent lipid overload disrupts the balance between PPARα–nuclear factor κB (NF-κB) signaling, resulting in NF-κB–dominant pro-inflammatory activity and early induction of the senescence-associated secretory phenotype (SASP). Concurrent impaired mitochondrial adaptation, increased oxidative stress, and reduced FAO promote p16 stabilization, reinforcing the senescent phenotype. Senescence-associated metabolic rewiring enhances CD36-mediated lipid uptake, thereby establishing self-amplifying feedback loops linking lipid accumulation, SASP signaling, and cellular senescence, which promote MASLD progression toward MASH.

Based on the available evidence, we propose a mechanistic framework in which hepatic lipid overload initially elicits a PPARα-dependent adaptive response that enhances mitochondrial βoxidation and limits LD accumulation. However, sustained metabolic overload progressively overwhelms this adaptive program, leading to mitochondrial dysfunction and excessive ROS production. Elevated mitochondrial ROS, together with a shift toward NF-κB dominant signaling, promotes early SASP induction and p16INK4a-mediated stabilization of the senescent state. The resulting accumulation of senescent hepatocytes, characterized by enhanced CD36-mediated lipid uptake, further amplifies the hepatic lipid burden and sustains inflammatory signaling, thereby driving MASLD progression (Figure 3).

Importantly, this conceptual model produces several testable hypotheses that can guide future research. First, we suggest that the timing and reversibility of CS induction may influence whether PPARα-driven FAO remains protective or becomes irreversibly suppressed, thereby promoting LD accumulation and chronic inflammation. Longitudinal studies combining senescence markers with metabolic flux analyses in experimental MASLD models will be essential for understanding this temporal transition.

Second, the identification of mitochondrial ROS and NF-κB as a key convergence point suggests that these pathways act as critical checkpoints through which ongoing lipid overload is converted into senescence stabilization. Therapeutic strategies aimed at restoring mitochondrial flexibility or reducing NF-κB–driven SASP signaling may therefore be promising approaches to prevent the shift from steatosis to MASH.

Third, the consistent association among CS stabilization, CD36 upregulation, and LD expansion indicates that senescent hepatocytes exhibit a distinct lipid uptake and storage program compared with proliferative hepatocytes under similar steatotic conditions. Single-cell and spatial transcriptomic approaches, combined with lipidomic profiling, will be crucial to clarify this heterogeneity and determine whether senescent hepatocytes constitute a metabolically specialized subpopulation that disproportionately drives disease progression.

Finally, given that SIPS and RS coexist in advanced MASLD, future studies should elucidate how these senescence pathways interact over time and whether their functional contributions differ across disease stages and metabolic contexts. Clarifying these interactions may facilitate the development of stage-specific senotherapeutic or metabolic interventions tailored to MASLD progression.

## Data Availability

No new data were created or analyzed in this study. Data sharing is not applicable to this article.

## Abbreviations

The following abbreviations are used in this manuscript:

ATM	Ataxia-telangiectasia mutated
CD36	Cluster of differentiation 36
CS	Cellular senescence
DDR	DNA damage response
ECM	Extracellular matrix
ER	Endoplasmic reticulum
EVs	Extracellular vesicles
FA	Fatty acid
FAO	Fatty acid oxidation
FASN	Fatty acid synthase
FFA	Free fatty acid
HCC	Hepatocellular carcinoma
HGF	Hepatocyte growth factor
IL	Interleukin
LD	Lipid droplet
MASH	Metabolic dysfunction-associated steatohepatitis
MASLD	Metabolic dysfunction-associated steatotic liver disease
mTOR	mammalian target of rapamycin
NAFLD	Non-alcoholic fatty liver disease
NF-κB	Nuclear factor kappa B
PPARα	Peroxisome proliferator-activated receptor alpha
ROS	Reactive oxygen species
RS	Replicative senescence
SA-β-gal	Senescence-associated β-galactosidase
SADF	Senescence-associated DNA damage foci
SAHF	Senescence-associated heterochromatin foci
SASP	Senescence-associated secretory phenotype
SIPS	Stress-induced premature senescence
TGF-β	Transforming growth factor beta
TG	Triglyceride
PLINs	Perilipins
